# Clinical Biochemical Indicators and Intestinal Microbiota Testing Reveal the Influence of Reproductive Age Extending from the Mother to the Offspring

**DOI:** 10.1128/spectrum.01076-22

**Published:** 2022-08-22

**Authors:** Xiaoqing Li, Bing Zhang, Jiayong Zheng, Haiying Chen, Jianqiong Zheng, Xiaomin Xu, Wenhuan Wang, Congcong Sun, Wenjing Lin, Kaixin Chen, Zitong Xu, Hongping Zhang, Jinfeng Wang

**Affiliations:** a Wenzhou People's Hospital/Wenzhou Maternal and Child Health Care Hospital/The Third Clinical Institute Affiliated to Wenzhou Medical University /The Third Affiliated Hospital of Shanghai University, Wenzhou, Zhejiang, China; b College of Food Science & Nutritional Engineering, China Agricultural University, Beijing, China; c University of Chinese Academy of Sciences, Beijing, China; d Beijing Institutes of Life Science, Chinese Academy of Sciences, Beijing, China; Temasek Life Sciences Laboratory

**Keywords:** childbearing age, lying-in woman, newborn, clinical biochemical assay, gut microbiome

## Abstract

Age is an important factor that determines the physiological functions of the human body, but the changes in maternal physiology, biochemistry, and intestinal flora related to reproductive age and their impact on offspring are not clear. Here, we tested and analyzed the clinical physiological and biochemical indicators and/or intestinal flora, matching the data of 252 parturients and their newborns. We found that 4 clinical indicators, including the white blood cell count and the absolute value of monocytes, were significantly related to the reproductive age (*P* < 0.05). The composition of the intestinal flora also varied with age, and the intestinal flora of advanced-age women (≥35 years old) was different from that of middle-aged women (>25 and <35 years old). We also found that changes in maternal clinical physiological and biochemical indicators related to reproductive age could reflect changes in the abundance of bacteria, such as *Peptococcus* and *Vibrio*, and changes in the intestinal flora spread to offspring. These results provide new evidence to explain the increased adverse pregnancy outcomes of mothers of inappropriate age, describe the increased health risks of newborns, help us examine the importance of age-appropriate birth from a broader perspective, and contribute to the discovery of mother-child bonds for a better understanding of healthy reproduction.

**IMPORTANCE** In this study, we demonstrated that physiological indicators and the gut microbiome fluctuate drastically among parturients of different reproductive ages and that there is a significant correlation between the two changes. Mothers of different ages had different gut microbes, and the gut microbiota varied as the childbearing age became too high. Changes in the gut microbiome with maternal reproductive age affected the offspring, and the influence of reproductive age on the intestinal flora had a synergistic effect between mother and child that was revealed for the first time. The maternal childbearing age might affect the colonization of the offspring's initial flora. The results provide new evidence to explain the increased adverse pregnancy outcomes of mothers of inappropriate age, describe the increased health risks of newborns, and contribute to the discovery of mother-child bonds for a better understanding of healthy reproduction.

## INTRODUCTION

Age-appropriate childbearing has always been a common focus of global concern. According to statistics, among women who gave birth from 2006 to 2015, 2.5% were 15 to 17 years old, 24.9% were 30 to 34 years old, and 14.9% were over 35 years old (1). A 2015 United Nations Report pointed out that with increasing social competition pressure, more professional women, changing fertility concepts, and the increasing maturity of assisted reproductive technology, over the past 30 years, the average age of childbearing in developed countries has risen globally; for example, the average age of childbearing increased from 26.5 years in 1980 to 29.4 years in 2010 in Europe (2). In particular, the proportion of elderly pregnant women in the world (generally, women who are 35 years old or older) shows a continuous upward trend (3). According to a report by the United States Centers for Disease Control, the proportion of first-time births to older women rose 23% from 7.5% in 2009 to 9.1% in 2014 (4). Meanwhile, although the fertility rate of women who give birth at a young age (under the age of 20) has dropped significantly in recent years (5, 6), incidence remains at 11% on a global scale, and 90% of low-age births occur in low-income countries, such as Nepal (7). Inappropriate childbearing is a common phenomenon worldwide.

Age is one of the important factors that determines the function of the human body. Maternal physical status and functional problems caused by too high or too low of a childbearing age will bring a series of adverse consequences, and the risk is the highest for those over 40 years old, followed by those between 35 and 39 years old and those between 15 to 17 years old (1, 8, 9). Compared with middle-aged women, adverse pregnancy outcomes, such as preeclampsia, postpartum hemorrhage, and gestational diabetes, are more common in women of higher age (1), and fetal miscarriage, premature delivery, and mortality are also increased (10). Among pregnant women 40 years of age and older, 10.82% experienced one or more adverse pregnancy outcomes, while the proportion for pregnant women aged 20 to 34 was 5.46% (11). Compared with mothers aged 20 to 24, teenage mothers aged 10 to 19 have an increased risk of eclampsia, puerperal endometritis, systemic infections, low birth weight, premature delivery, and severe neonatal diseases (6). Both premature births and older births increase the risk of maternal and infant health. This may be caused by many factors, such as premature or advanced age, lower body functions, unstable hormone levels, and human microbial disorders (12). Although the reasons are not yet clear, it is certain that the harm caused by bearing a child out of age has become a huge threat to both human health and population quality.

Through the detection of clinical biochemical indicators, the diagnosis, prediction, and monitoring of the health and the physiological state of the parturient during pregnancy can be realized, and these indicators usually include, but are not limited to, the detection of various proteins, enzymes, carbohydrates, lipids, and multiple ions in blood or urine, which are used to reflect liver function, kidney function, blood lipids, glucose metabolism, the thyroid, and other aspects of the body (13). Similarly, human symbiotic microorganisms can also be used as biomarkers to reflect maternal health status during pregnancy (14). Increasing evidence shows that the composition of the intestinal flora of pregnant women depends on the kidney status (15), heart status (16), thyroid hormone level (17), blood pressure level (18), blood sugar level (19), degree of obesity (20), dietary composition (21), etc. With increasing age, intestinal microbes continue to change (22–25), and an age-related flora imbalance promotes intestinal permeability, systemic inflammation, and abnormal macrophage function (26). As a result, individuals gradually acquire disease-related flora characteristics and gradually lose health-related flora characteristics (27). Although some studies have pointed out that changes in serum physiological and biochemical indicators during pregnancy are related to intestinal microbes (19), there is no research on the relationship between reproductive age, physiological and biochemical indicators, and intestinal flora. Therefore, it is necessary to use these clinical physiological, biochemical, and microbiological indicators to measure the impact of reproductive age on mothers and their offspring.

The childbearing age of Chinese women is increasing year by year. Especially in recent years, groups of elderly couples have new birth plans. They may face a series of reproductive health problems caused by a childbearing age that is too old. In view of this, this study analyzed the physiological and biochemical indicators and intestinal microbiomes of pregnant women of different ages to reveal the relationship between age, age-related clinical indicators, and changes in the flora. This study further explored the influence of reproductive age on the initial intestinal flora of newborns. On this basis, the potential impact of colonization clarifies the importance of the reproductive age to the physiology of pregnancy and the health of offspring. This is expected to generate a new understanding in the field of reproductive health.

## RESULTS

A total of 252 parturients and newborns were recruited in this study. To explore the changes in the physical functions and the intestinal microbes in women of different ages, pregnant women were divided into groups with a span of 5 years according to their ages, including a 20 to 25-year-old group, a 25 to 30-year-old group, a 30 to 35-year-old group, and a >35-year-old group (Fig. S1A). We obtained clinical data, including physiological and biochemical indicators of healthy parturients of different ages, and collected their feces and the meconium (the first excrement) of their newborns. Each sample was sequenced for the V3-V4 region of the 16S rRNA gene, with an average of 194,509 reads per sample (Fig. S1B and C).

### Physiological indicators fluctuate drastically among parturients of different reproductive ages.

General clinical laboratory examinations and the detection of physiological and biochemical indicators are important parts of the management of pregnant women, as they provide objective data for clinical decision-making in various situations, including inflammatory state detection, metabolic index detection, liver function detection, renal function detection, and thyroid function detection (13). Age was an important factor affecting these indicators (28, 29). To explore the influence of childbearing age on maternal physiological status, we first analyzed the clinical background data of pregnant women in late pregnancy, including their physical examination results, disease history, dietary habits, medication history, etc., and we then analyzed the results of physical examinations of their newborns (Table S1). With increasing age, the number of pregnancies and abortions of pregnant women increased significantly, and the body mass indices (BMI) and weights of the newborns were also different (false detection rate [FDR] < 0.05; analysis of variance [ANOVA]).

Next, we detected the clinical physiological and biochemical indicators of pregnant women in different age groups, including the biochemical indicators of peripheral blood, routine blood tests, blood coagulation-related indicators, thyroid indicators, and routine urine indicators, and grouped them according to four age groups for statistical analysis (Table 1; Table S2). It was found that the platelet distribution width, white blood cell count, absolute neutrophil count, and absolute value of monocytes displayed significant changes in each group (FDR < 0.05). It could be seen that with the increase of age, the platelet distribution width showed a trend of significant increase after decreasing, while the values of the white blood cell count, the absolute count of neutrophils, and the absolute value of monocytes showed an opposite trend of increasing first and then decreasing. These findings suggested that the different changes in these physical function indices during pregnancy might be related to reproductive age.

**TABLE 1:** Biochemical indicators and routine blood indicator information of participants in different age groups^*a*^

Category	Variables	20 to 25 y (N = 17)	25 to 30 y (N = 33)	30 to 35 y (N = 36)	≥35 y (N = 48)
Biochemical indices	Total protein (g/L)	63.05 ± 5.79	62.86 ± 6.41	60.88 ± 6.37	58.80 ± 6.84
	Creatinine (μmol/L)	44.59 ± 6.53	42.6 ± 6.47	41.64 ± 6.45	46.26 ± 8.21
	Total bilirubin (μmol/L)	8.48 ± 2.44	8.15 ± 3.98	8.69 ± 3.29	8.74 ± 3.29
	Direct bilirubin (μmol/L)	1.45 ± 0.43	1.87 ± 2.66	1.3 ± 0.37	1.30 ± 0.48
	Indirect bilirubin (μmol/L)	7.02 ± 1.37	6.26 ± 2.36	7.03 ± 1.82	6.91 ± 2.55
	Albumin (g/L)	30.9 ± 2.403	31.08 ± 1.87	31.15 ± 2.97	29.40 ± 2.62
	Globulin (g/L)	27.95 ± 2.89	28.47 ± 3.44	27.03 ± 3.32	26.27 ± 3.18
	Ratio of albumin to globulin	1.11 ± 0.06	1.10 ± 0.12	1.16 ± 0.12	1.13 ± 0.14
	Alanine transaminase (U/L)	17.47 ± 6.50	15 ± 7.16	14.27 ± 7.74	13.72 ± 7.31
	Aspartate aminotransferase (U/L)	16.33 ± 2.66	16.39 ± 2.97	16.10 ± 2.57	16.94 ± 5.83
	Alkaline phosphatase (U/L)	209.17 ± 84.83	195.67 ± 47.50	183.62 ± 59.52	184 ± 49.10
	Gamma-glutamyl transferase (U/L)	10.83 ± 2.93	14.06 ± 10.26	11.43 ± 6.96	10.63 ± 4.70
	Total bile acid (μmol/L)	3.78 ± 3.19	2.71 ± 1.72	2.50 ± 1.26	3.09 ± 2.65
	Cholyglycine (μmol/L)	4.58 ± 0.88	4.39 ± 1.28	3.86 ± 0.51	4.33 ± 1.70
	Fibronectin (mg/L)	186.83 ± 27.47	222.76 ± 28.57	212.62 ± 35.23	202.4 ± 33.94
	Glycated hemoglobin (%)	5.34 ± 0.14	5.47 ± 0.28	5.54 ± 0.43	5.59 ± 0.32
	Glucose (mmol/L)	5.38 ± 1.41	4.50 ± 1.05	4.58 ± 1.18	4.55 ± 1.07
	Urea (mmol/L)	3.04 ± 0.81	3.03 ± 0.73	3.02 ± 0.89	3.16 ± 0.76
	Retinol binding protein	40.83 ± 6.05	44.5 ± 4.67	42.5 ± 4.96	41.22 ± 5.57
	β2 microglobulin (mg/L)	1.83 ± 0.1169	1.99 ± 0.22	1.86 ± 0.1376	1.99 ± 0.2978
	Uric acid (μmol/L)	334.19 ± 98.61	322.74 ± 70.61	326.67 ± 86.38	327.82 ± 74.27
	Triglyceride (mmol/L)	2.57 ± 0.90	2.69 ± 0.53	3.26 ± 1.36	3.57 ± 2.78
	Total cholesterol (mmol/L)	6.29 ± 0.77	6.29 ± 1.24	6.22 ± 1.36	6.34 ± 1.52
	High density lipoprotein cholesterol (mmol/L)	1.66 ± 0.40	1.74 ± 0.38	1.71 ± 0.37	1.63 ± 0.27
	Low density lipoprotein cholesterol (mmol/L)	3.82 ± 0.85	3.42 ± 0.79	3.40 ± 1.12	3.50 ± 0.87
	Non high density lipoprotein cholesterol (mmol/L)	4.63 ± 1.02	4.55 ± 0.94	4.52 ± 1.17	4.70 ± 1.33
	Lipoprotein(a) (mg/dL)	20.67 ± 12.42	14.47 ± 10.57	15.86 ± 12.38	16.03 ± 11.26
	Free fatty acid (μmol/L)	271.17 ± 84.38	338.76 ± 153.00	347.95 ± 119.05	362.53 ± 157.47
	Homocysteine (μmol/L)	7.23 ± 2.64	7.35 ± 1.50	7.62 ± 3.23	7.35 ± 1.99
	Hypersensitive C-reactive protein (mg/L)	1.85 ± 0.96	2.95 ± 1.76	4.68 ± 5.40	3.2 ± 2.14
	K (mmol/L)	3.68 ± 0.30	3.80 ± 0.26	3.81 ± 0.21	3.78 ± 0.21
	Na (mmol/L)	136.35 ± 1.17	136.33 ± 1.79	136.30 ± 1.65	136.40 ± 1.75
	Cl (mmol/L)	105.35 ± 1.46	105 ± 2.53	105.64 ± 1.67	106.64 ± 5.05
	P (mmol/L)	1.39 ± 0.16	1.23 ± 0.14	1.24 ± 0.16	1.24 ± 0.17
	Ca (mmol/L)	2.18 ± 0.12	2.15 ± 0.10	2.15 ± 0.10	2.14 ± 0.13
	Mg (mmol/L)	0.74 ± 0.05	0.78 ± 0.04	0.76 ± 0.04	0.77 ± 0.06
	Fe (μmol/L)	8.67 ± 4.66	13.57 ± 6.62	12.19 ± 4.06	13.95 ± 12.71
	Isoenzymes of creatine kinase (U/L)	8.33 ± 1.63	11.29 ± 4.81	9.86 ± 2.69	9.7 ± 5.55
	Lactate dehydrogenase (U/L)	169.5 ± 16.45	180.47 ± 33.67	175.24 ± 34.67	180.8 ± 44.01
	Amylase	83.64 ± 18.79	90.17 ± 18.32	90.33 ± 21.80	79.71 ± 17.77
	Cholinesterase	5403.27 ± 695.85	5511.25 ± 762.20	5301 ± 762.80	5673.2 ± 763.94
	Lactic	2.62 ± 1.13	2.58 ± 0.74	2.27 ± 0.51	2.21 ± 0.57
	Serum amyloid A	4.16 ± 1.93	5.44 ± 2.17	10.83 ± 17.27	7.65 ± 7.85
	Creatine Kinase (U/L)	39.82 ± 25.08	49.96 ± 28.16	58.53 ± 52.97	59.73 ± 90.75
	Blood ammonia	18.13 ± 4.82	20 ± 6.93	17.18 ± 5.42	16.4 ± 6.52
	Mass determination of creatine kinase isoenzyme	1.31 ± 0.65	1.92 ± 1.64	1.45 ± 0.65	1.59 ± 0.75
	Procalcitonin determination	0.04 ± 0.02	0.038 ± 0.01	0.04 ± 0.03	0.04 ± 0.01
	C-reactive protein	4.45 ± 3.99	2.9 ± 2.14	4.62 ± 5.37	3.52 ± 2.91
	Vitamin D	27.07 ± 13.65	22.3 ± 13.83	27.01 ± 10.35	29.21 ± 8.94
Blood routine test indices	White blood cell count (10^9^/L)^*b*^	7.94 ± 2.30	8.94 ± 2.08	8.28 ± 1.95	7.37 ± 1.65
	Absolute neutrophil count (10^9^/L)^*b*^	5.94 ± 2.15	6.61 ± 1.72	6.03 ± 1.65	5.38 ± 1.48
	Absolute value of monocytes (10^9^/L)^*b*^	0.47 ± 0.14	0.65 ± 0.22	0.62 ± 0.20	0.50 ± 0.14
	Platelet distribution width (fl)^*b*^	15.42 ± 2.04	15.03 ± 2.36	13.5 ± 2.51	14.77 ± 2.56
	Percentage of neutrophils (%)	73.42 ± 6.75	73.53 ± 4.53	72.23 ± 5.25	72.1 ± 6.10
	Percentage of lymphocytes (%)	19.5 ± 6.11	18.24 ± 4.04	19.00 ± 4.80	19.61 ± 5.15
	Percentage of monocytes (%)	6.18 ± 1.51	7.21 ± 1.47	7.53 ± 1.91	6.99 ± 1.69
	Percentage of eosinophils (%)	0.75 ± 0.74	0.86 ± 0.79	1.12 ± 1.05	1.18 ± 1.87
	Percentage of basophilic granulocyte (%)	0.15 ± 0.09	0.15 ± 0.16	0.12 ± 0.11	0.12 ± 0.12
	Absolute value of lymphocyte (10^9^/L)	1.46 ± 0.32	1.58 ± 0.3693	1.52 ± 0.3498	1.40 ± 0.2782
	Absolute value of eosinophils (10^9^/L)	0.06 ± 0.07	0.08 ± 0.09	0.09 ± 0.09	0.08 ± 0.15
	Red blood cell count (10^12^/L)	4.04 ± 0.24	4.06 ± 0.39	3.99 ± 0.33	3.99 ± 0.40
	Hemoglobin (g/L)	120.94 ± 8.11	121.69 ± 10.37	119.86 ± 10.48	118.68 ± 13.95
	Hematokrit (%)	36.59 ± 1.62	36.41 ± 2.97	35.71 ± 2.83	35.62 ± 3.72
	Mean corpuscular vol (fl)	90.69 ± 5.14	90.20 ± 7.32	89.78 ± 5.34	89.64 ± 7.13
	Mean corpuscular hemoglobin (pg)	29.96 ± 2.20	30.15 ± 2.62	30.13 ± 1.97	29.87 ± 2.81
	Mean corpuscular hemoglobin concn (g/L)	330.29 ± 12.51	334.28 ± 11.88	335.5 ± 9.41	332.85 ± 12.74
	Red blood cell distribution width (%)	13.84 ± 1.25	16.99 ± 9.66	14.37 ± 1.93	14.95 ± 4.89
	Platelet count (10^9^/L)	187.24 ± 31.04	195.56 ± 52.65	199.78 ± 45.40	185.09 ± 46.56
	Thrombocytopenia (%)	0.18 ± 0.03	0.20 ± 0.05	0.20 ± 0.04	0.19 ± 0.04
	Mean platelet vol (fl)	9.86 ± 0.98	10.26 ± 1.22	10.12 ± 0.94	10.53 ± 1.15
	Platelet large cell ratio (%)	26.48 ± 6.88	28.42 ± 7.08	26.55 ± 6.50	29.96 ± 7.63

a The *P* values were adjusted with the Benjamini-Hochberg correction.

b Denotes adjusted *P* < 0.05.

### Maternal gut microbiome varied with reproductive age.

We examined the relationship between maternal age and the gut microbiome to extensively evaluate the impact of reproductive age on maternal physiology and to look for biomarkers that reflect the health status of pregnancy. First, we treated age as a continuous variable and analyzed the changes in intestinal microflora in pregnant women. With increasing age, we found that there was no significant correlation between maternal age and α-diversity (Fig. S2A–F).

**FIG 1:**
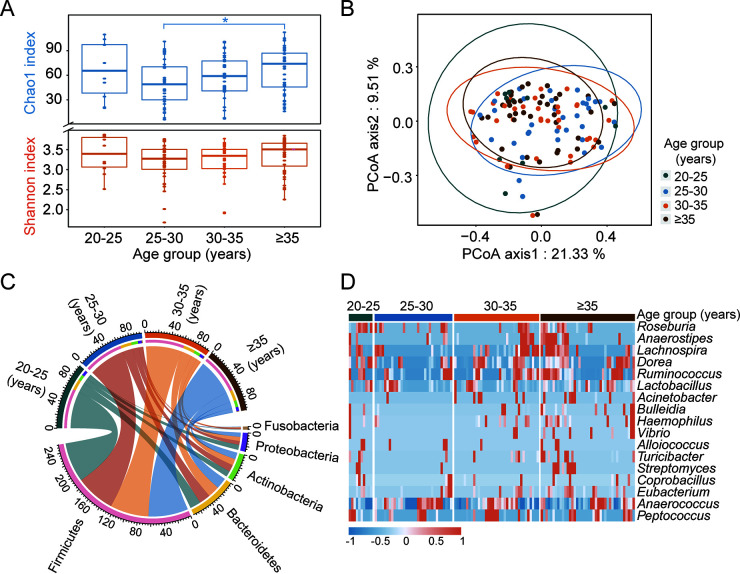
Significant changes in the gut microbes of elderly women. (A) Comparison of the Chao1 index and the Shannon index in maternal gut microbiota for different age groups. (B) PCoA for different age groups. (C) The top 5 most abundant phyla in maternal gut microbiota. (D) Bacteria with statistically significant differences between different age groups at the genus level. *, *P* < 0.05.

Then, we divided the ages into four groups with a span of 5 years to check the fecal flora across the different age groups. The results showed that the Shannon, Chao1, ACE, richness, and Simpson indices of the gut microbiota in the elderly group (≥35 years old) were higher than those in the other age groups, while the Pielou index was the opposite (Fig. 1A; Fig. S3A–D). When a correlation analysis was conducted among women aged over 25 years, it was found that with increasing age, the microbial diversity of the community increased significantly (*P < *0.05; Spearman correlation analysis) and that the evenness decreased gradually (Fig. S4A–F). Compared with the parturients of the 25 to 30 and 30 to 35-year-old groups, the diversity and evenness of gut microbes in the 20 to 25-year-old group were more similar to those of women aged ≥35 (Fig. 1A; Fig. S3A–D). To further measure the impact of reproductive age on maternal intestinal flora, we performed a principal coordinates analysis based on the Bray-Curtis (BC) distance. As a result, the microbial communities between individuals showed a trend of gradual aggregation with increasing age (Fig. 1B), although a permutational analysis of variance (PERMANOVA) did not show significant differences. These results indicate that mothers of different ages might have different gut microbes and that the structure of the gut microbiota changed when the childbearing age was too high.

In the intestinal flora of the parturient, the proportion of the top three bacteria with the highest relative abundances, namely, Firmicutes, Bacteroidetes, and Actinobacteria, had almost no difference among the different age groups (Fig. 1C; Fig. S5). However, Proteobacteria, whose proportion was 8.17% in the 20 to 25-year-old group, showed a trend of first decreasing and then increasing slightly with increasing age (*P = *0.795; ANOVA). Furthermore, a linear discriminant analysis effect size showed that the abundance of Haemophilus, *Eubacterium*, *Bulleidia*, *Streptomyces*, and *Turicibacter* in the elderly group (≥35 years old) increased significantly (Fig. 1D), suggesting that age-related changes in physical function might be related to these bacteria.

### Maternal clinical indicators significantly correlated with intestinal microbes.

**FIG 2:**
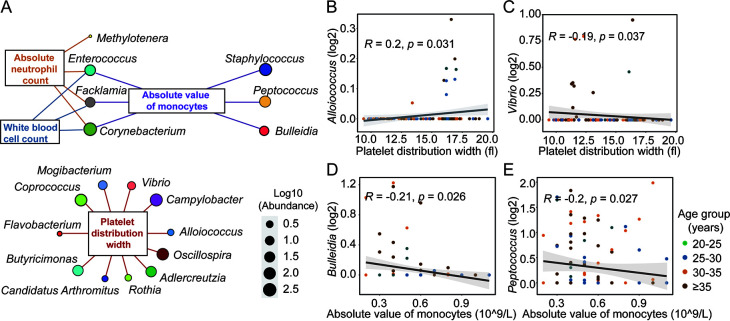
The clinical physiological and biochemical indices of puerperae related to intestinal microbes. (A) All bacteria that were significantly correlated with clinical test indicators, including platelet distribution width, white blood cell count, absolute neutrophil count, and absolute value of monocyte indicators. (B) Correlation between platelet distribution width and *Alloiococcus.* (C) Correlation between platelet distribution width and *Vibrio*. (D) Correlation between the absolute value of monocytes and *Bulleidia*. (E) Correlation between absolute value of monocytes and *Peptococcus*. Correlation was calculated via the Spearman method.

To explore the relationship between the clinical indicators related to reproductive age and the intestinal flora of pregnant women, we conducted a correlation analysis between four clinical physiological and biochemical indicators and four general clinical indicators that had significant differences across the different age groups and the intestinal microflora of pregnant women. A Spearman correlation analysis with bacteria at the genus level showed that these indices and age were both significantly correlated with the relative abundance of some genera (*P* < 0.05; Spearman correlation analysis) (Fig. 2A–E; Fig. S6A–H). Among them, platelet distribution width had the closest and most complex relationship with the bacteria.

To further measure the relationship between the characteristic bacteria in different age groups (Fig. 1D) and the bacteria strongly associated with the clinical indicators (Fig. 2A), we searched for the overlap of these bacteria (Fig. S7A) and found that *Bulleidia*, *Vibrio*, *Lactobacillus*, *Alloiococcus*, *Peptococcus*, and *Lachnospira* cooccurred. Of them, *Vibrio* bacteria associated with the platelet distribution width, and *Bulleidia* bacteria associated with the absolute value of monocytes. Both were also significantly related to age (Fig. S7B). We then calculated the correlation value for each pair of clinical indicator and microbe to get an empirical distribution of associations. Then, we obtained the associations from the empirical null distribution (Table S3). This showed that there were 16 bacteria, including the *Bulleidia* bacteria associated with the absolute value of monocytes and the *Vibrio* bacteria associated with the platelet distribution width, that were roughly the same as those found by the above method. In addition, we also found 8 new bacteria, including *Dorea* and *Ruminococcus*, that were associated with clinical physiological and biochemical indicators.

It is worth noting that there were no identical bacteria among all the bacteria significantly related to the four general clinical indicators of pregnancy, including pregnancy history, abortion history, miscarriage history, and BMI. No age-related bacteria overlapped the same bacteria, indicating that although the 4 general clinical indicators were significantly different among these age groups (Table S1), they may not largely affect the age-related flora.

### Changes in the gut microbiome with maternal reproductive age extend to the offspring.

**FIG 3:**
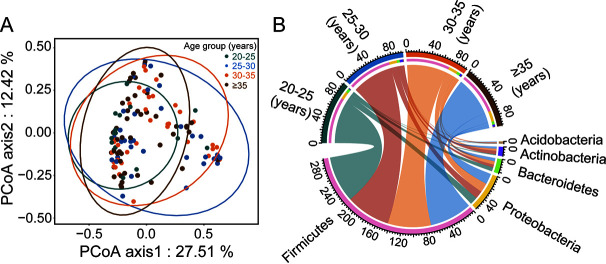
Significant changes occurred in the fecal microbiota of infants with young or advanced maternal age. (A) PCoA for meconium flora of newborns in different age groups. (B) The top 5 most abundant phyla in neonatal meconium.

To investigate the potential effect of reproductive age on offspring, we analyzed neonatal meconium samples. The results showed that although there was no significant difference in the α-diversity of the meconium samples from different age groups (Fig. S8A–F), according to the PCoA results, among women over 25 years old, the community similarity of intestinal microflora among newborns increased with increasing maternal childbearing age (Fig. 3A). Compared with the composition of the intestinal flora of mothers (Fig. 1C), the abundance of dominant bacterial phyla varied greatly among the different age groups in the neonatal meconium (Fig. 3B). The proportion of Actinobacteria showed significant differences between the different age groups (*P = *0.034; ANOVA) (Fig. S9). With the increase of reproductive age, the proportion of Firmicutes bacteria continued to decrease (77.33% in 20 to 25 years old, 74.09% in 25 to 30 years old, 72.33% in 30 to 35 years old and 70.58% in ≥35 years old [*P = *0.637; ANOVA]), while the proportion of Proteobacteria showed an opposite, increasing trend (Fig. S9).

**FIG 4:**
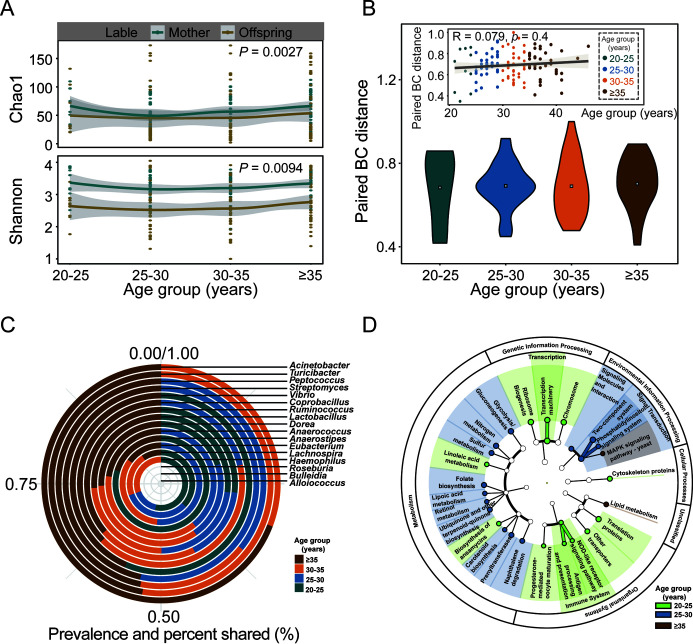
The fecal microbes of the parturient covaried with the initial flora of the fetus. (A) Comparison of the Chao1 index and the Shannon index of bacterial communities for different age groups in mother feces and matched newborn meconium samples. (B) Comparison of Bray-Curtis distances of bacterial communities for different age groups in mother feces and matched newborn meconium samples. (C) The transmission of bacteria with significant differences between different age groups at the genus level. (D) Prediction of the functions of different microflora in meconium samples from different age groups by PICRUSt.

To explore whether the influence of reproductive age on the intestinal flora has a synergistic effect between mother and child, we analyzed samples of mothers and their paired newborns. The Chao1 index and the Shannon index between mother and child had similar trends and had a significant correlation (*P* < 0.05; Wilcoxon test) (Fig. 4A). The ACE index, richness index, and Pielou index were all significantly correlated (*P < *0.05, Wilcoxon test) (Fig. S10A–D), indicating that the microbial diversity of the fecal flora between mother and child had the same changing trend. The BC distance between mother-child paired samples showed no significant correlation with increasing age, and there was no significant difference between the BC distances of different age groups (Fig. 4B), indicating that changes in the fecal flora of the mother were synchronized with changes in the fecal flora of the offspring. The bacteria with significant differences associated with different age groups of mothers were transmitted to the paired offspring more in the older age groups than in the younger age groups (Fig. 4C).

To explore the relationship between the initial colonization of the offspring flora and the clinical indicators related to maternal age, we conducted an association analysis between four clinical indicators and all of the bacterial genera in the neonatal meconium (Fig. S11A). The results showed that these age-related maternal clinical indicators significantly affected the neonatal meconium (*P* < 0.05; Spearman correlation analysis). *Corynebacterium* and *Bulleidia* are bacteria of intestinal microbiota that are related to clinical physiological and biochemical indices in both mother and offspring (Fig. S11B). It was speculated that a change in maternal age might affect the colonization of the offspring's intestinal microbiome through these clinical indicators. Furthermore, we used PICRUSt to predict the function of the differential flora in the different age groups (Fig. 4D). Compared with the other age groups, the metabolism and environmental information processes were most active in the 25 to 30-year-old group. Genetic information processing, cellular processing, and organismal system activities were dominant in the 20 to 25-year-old group, while lipid metabolism activities were stronger in the ≥35-year-old group, suggesting that age-related bacteria might affect progeny through these pathways.

## DISCUSSION

Age has a significant impact on clinical physiological and biochemical indicators. As age increases, physiological functions decline. The intuitive performance is that physiological and biochemical indicators, including proteins, metabolites, and other biomolecules, will change (28, 29) and that health conditions will deteriorate (30). This may be because age-related vascular aging can cause arterial degeneration and hardening, thereby impairing vascular function and ultimately leading to damage to organs, such as the heart, brain, kidney, and liver (31). Damage to these organs is closely related to changes in the intestinal microbiota in puerperae (15, 17, 18). However, the relationship between changes in clinical physiological and biochemical indicators that reflect body function and intestinal microbes caused by age is still unclear. Our study found that the physiological and biochemical indicators and the intestinal microbiomes of women of different reproductive ages fluctuated drastically. The maternal clinical physiological and biochemical indicators related to reproductive age will change. These indicators had a significant correlation with intestinal microbes, which provided important evidence to explain the relationship between age, organism status, and the gut microbiome.

In this study, we found that the bacterial number and diversity of the gut microbiota in pregnant women showed a U-shaped trend. In the groups over 25 years old, it showed an upward trend with increasing age, and it decreased in the groups between 20 and 30 years old, with an inflection point at approximately 25 to 30 years old. Interestingly, studies have found that, compared with children of mothers who were too young or too old, mothers and newborns of mothers of moderate childbearing age had a lower risk of adverse pregnancy outcomes. Age had an inverted U-shaped relationship with the incidence of adverse pregnancy outcomes; the preterm birth rate was the lowest for mothers 25 to 29 years old (32), and newborns born to the youngest or oldest mothers had lower birth weights than did those born to mothers aged 30 to 37 years (33). With increasing age, the fertility of women also showed an inverted U-shaped change, and women of a childbearing age that was either too low or too high showed low fertility. The best childbearing age of the population curve was between 25 and 35 years old, and the optimal U-shaped synthesis point was 25.3 years of age, which is basically in line with the human fertility curve (34).

Compared with the relatively stable or even decreasing fertility in the younger age group, the number of elderly births showed an increasing trend, year over year (3). Especially with the changes in China's social environment, the age of pregnant and lying-in women continued to increase, and the rate of advanced-age women increased from 8.52% in 2013 to 15.82% in 2017 (35). Compared with pregnant women of middle age, the physical, psychological, and social factors of older women were special, and their pregnancy complications, such as stillbirth, gestational diabetes, and other diseases, increased significantly (36). This suggests that we need to improve prepregnancy check-ups for older women and the prevention of adverse pregnancy outcomes for older women. Biomarker mining and the prediction of adverse pregnancy outcomes through microbial and clinical indicators is a new strategy. Many studies have used machine learning methods to predict adverse pregnancy outcomes. When combined with *Lautropia* and *Neisseria* bacteria in dental plaque and Streptococcus in saliva, the area value under the curve reached 0.83, and the maximum value reached 0.89 (14). Stillbirth was better predicted by age, parity, and previous adverse pregnancy outcomes (37). In this study, it was found that changes to several clinical indicators related to the childbearing age of the parturient could reflect changes in maternal bacterial abundance and changes in the intestinal flora spread to the offspring. This suggested that we might be able to find suitable predictive markers to avoid adverse pregnancy outcomes through the age, clinical physiological and biochemical indicators, and microbes of advanced-age women.

How to manage pregnancy and the perinatal period of nonsuitable age parturients, especially elderly parturients, also deserves attention. Studies have shown that changing the gut microbiome is a potential new strategy to fight aging, and some age-related factors, such as diet, lifestyle, inflammation, etc., could worsen the steady-state of the interactions between gut microbes and the host, leading to a shortening of the lifespan. Exercise and dietary interventions could alleviate age-related changes in the body and maintain a healthy gut microbiota to combat pathological and physiological processes related to human aging (38). Different dietary interventions could change the composition of *Eubacterium*, *Ruminococcus*, *Lachnospira*, and *Veillonella* bacteria (39) and improve aging (40). Changing diet could not only affect the intestinal microbes of the parturient but also have an important impact on the metabolic status of newborns and the intestinal microbiota; through vertical transfer, the maternal diet might affect the baby’s gut microbiota. For example, maternal fruit intake was related to the structure of the baby’s intestinal microbial community; the increase of fruit intake was related to the ratio of Streptococcus/*Clostridium* in natural birth babies, and dairy intake was also associated with an increased chance of high *Clostridium* aggregation in babies born by C-section (41). It was speculated that adding or reducing specific nutrients in the diet to target the inhibition/promotion of the growth of these harmful bacteria or other probiotics related to childbearing age could improve or correct the effect of childbearing age, meaning that we could use dietary interventions to manage advanced-age women during pregnancy.

In our study, we found that the number of gravidae, the number of pregnancies, and the abortion history were associated with changes in maternal microbes, which may also have a potential impact on maternal and child health. However, the bacteria associated with these indicators are different from those associated with age. Body mass index might also be an important factor in maternal gut microbes; compared with women at a normal weight, obese patients had gut microbiota that were enriched with bacteria of the Negativicutes class and the Proteobacteria phylum, such as *Megamonas*, *Succinatimonas* and *Dialister* (42). The different effects of bacteria associated with each of these factors on the host deserve further study in the future.

### Conclusion.

In conclusion, age-related clinical physiological and biochemical changes were associated with variations of maternal gut microbiota, and these changes were also associated with the colonization of offspring gut microbiota. The intestinal microbiota of puerperae and progeny changes synergistically with age, especially advanced age (≥35 years), which may cause changes in the intestinal flora and further affect the offspring. Our research provides more evidence from the perspective of clinical physiological and biochemical indicators and changes in the intestinal flora of mothers and newborns. The causal relationship between age-related clinical physiological and biochemical indicators and maternal intestinal microbiomes remains unresolved. However, there is no doubt that both clinical biochemical indicators and intestinal microbiota are related to maternal age. This will provide novel knowledge for the use of specific microbes to reflect clinical indicators and for exploring their corresponding physiological effects.

## MATERIALS AND METHODS

### Recruitment.

Parturients of different ages and their newborns were recruited from Wenzhou People’s Hospital. All of the volunteers were from Wenzhou People's Hospital. The study was approved by the Ethics Committee of Wenzhou People's Hospital (No. WR2021-215), and informed consent was obtained. All of the pregnant women were Han Chinese and local residents, with no history of smoking or drinking. All of the women were given a simple diet survey, and they ate three normal meals a day and were nonvegetarians. None of the pregnant women had received any drugs, antibiotics, or probiotics within 6 months. Patients with systemic and metabolic diseases, such as diabetes, hypertension, gestational diabetes, gestational hypertension, cardiovascular disease, tumors, infection, etc. were excluded.

### Sample collection.

Stool, peripheral blood, and urine from pregnant women in the third trimester (≥28 weeks and before delivery) and newborn meconium samples were collected according to the method described in our previous study (19). In brief, after a physical examination of all subjects, samples were collected by a professional doctor. Fecal samples of mothers preparing for cesarean section and meconium samples of their offspring within 48 h of birth were collected for a microbiome analysis, and peripheral blood samples of the mothers were collected during the same period for routine blood tests and analyses of biochemistry, coagulation function, and thyroid function. Urine samples were collected at the same time for routine urinalysis. All samples were stored in a −80°C refrigerator within 2 h for testing.

### Clinical laboratory tests.

Coagulation-related indicators were analyzed using an automatic coagulation analyzer (ACL-TOP700). Thyroid-related indicators were analyzed by a Beckman Coulter DXI 800 automatic luminescence immunoanalyzer, and total T4 and free T3 were detected by the chemiluminescence method. Biochemical indicators were analyzed by a Beckman Coulter AU 5800 automatic biochemical analyzer. A Unicel DxH800 Coulter cell analysis system was used for routine blood analysis. Urine was routinely analyzed using an FUS-200 automatic urine formed element analyzer.

### DNA extraction and 16S rRNA amplicon sequencing.

In a strictly controlled, separated, and sterile workplace, a QIAamp PowerFecal DNA Kit (Qiagen, Germany) was used to extract DNA from stool samples in accordance with the instructions. In brief, DNA was extracted from the fecal and meconium samples (0.5 g) by using a QIAamp PowerFecal DNA Kit (Qiagen, Germany) according to the manufacturer’s protocol. Subsequently, the V3 and V4 regions of the bacterial 16S rRNA gene were amplified by polymerase chain reaction (PCR), using the primers F3: CCTACGGGNBGCASCAG and R4: GACTACNVGGGTATCTAATCC. PCRs were performed in a 25 μL mixture containing 5 μL of 5 × GC Buffer, 0.5 μL of KAPA dNTP Mix, 0.5 μL of KAPA HiFi HotStart DNA polymerase, 0.5 μL of each primer (10 pM), and 50 to 100 ng of template DNA. PCR cycling included 95°C for 3 min, followed by 25 cycles at 95°C for 30 s, 55°C for 30 s, and 72°C for 30 s, with a final extension at 72°C for 5 min. The PCR cleanup used AMPure XP beads to purify the 16S V3 and V4 amplicons away from free primers and primer dimer species. PCRs were performed in a 25 μL mixture containing 5 μL of 5 × GC Buffer, 0.75 μL of KAPA dNTP Mix, 0.5 μL of KAPA HiFi HotStart DNA polymerase, 1.5 μL of each primer (10 pM), and 5 μL of purified product. PCR cycling included 95°C for 3 min, followed by 8 cycles at 95°C for 30 s, 55°C for 30 s, and 72°C for 30 s, with a final extension at 72°C for 5 min. The amplicons were subsequently purified by AMPure XP beads to clean up the final library before quantification. Finally, the purified amplicons were pooled in equimolar amounts and paired-end sequenced (2 × 250) on an Illumina MiSeq platform according to standard protocols.

### Bioinformatic processing, visual and statistical analysis.

Read processing was carried out in QIIME2 (version 2020.2.0). Reads were trimmed, pairs were merged and denoised, and representative sequences were chosen using DADA2 (43). Taxa were assigned to representative sequences by the QIIME2 feature classifier using the BLAST+ algorithm (44, 45), aligning sequences against the Greengenes version 13_8 99% similarity database. Bacteria that appeared less than 10 times in all abundance tables and had an absolute abundance value of less than 5 were eliminated. All analyses were performed in R version 4.1.0 within RStudio version 1.4.1717, and all figures were made using the ggplot and ggpubr packages.

Sample clustering based on the gut bacterial community was performed via a principal coordinate analysis, and differences between age groups were assessed via a PERMANOVA. After standardization, a partial least-squares discriminant analysis (PLS-DA) and a linear discriminant analysis (LDA) were used to identify the different bacterial genera in the different age groups. Genera identified as significantly different bacteria between groups (*P* < 0.05; Wilcoxon test) were further assessed by LDA using Galaxy (http://huttenhower.sph.harvard.edu/lefse/), and LDA scores of >2.0 were considered differential signatures. Bacterial function was also predicted through PICRUSt using Galaxy. The correlations between age, the clinical indicators, and the bacteria were calculated via the Spearman method. For analyses regarding multiple comparisons, we used the Benjamini-Hochberg method to correct for multiple testing.

### Data availability.

Sequencing data were deposited in Genome Sequence Archive (http://bigd.big.ac.cn/gsa) under the accession number HRA001924. All of the clinical data are anonymous and publicly available at https://github.com/callAgene/Supplementary_materials_XiaoqingLi_2022.
