# Supplementary material for: Clinical Biochemical Indicators and Intestinal Microbiota Testing Reveal the Influence of Reproductive Age Extending from the Mother to the Offspring
Source: Microbiol Spectr. 2022 Aug 22;10(5):e01076-22. doi: 10.1128/spectrum.01076-22 (PMC9602618; doi:10.1128/spectrum.01076-22)
Supplement: Supplemental file 1 — Tables S1 to S3 and Fig. S1 to S11. Download spectrum.01076-22-s0001.pdf, PDF file, 2.5 MB [file spectrum.01076-22-s0001.pdf]

Category	Variables		20-25 years (N=17)	25-30 years (N=33)	30-35 years (N=36)	⩾35 years (N=48)
General information	Ethnic Han (%)		100	100	100	100
	Living area (Wenzhou, China) (%)		100	100	100	100
	Caesarean section (%)		100	100	100	100
	Age (year)	*	22.41±1.50	27.36±1.52	32.17±1.30	37.92±2.59
	Gestational weeks (week)		38.89±1.27	38.75±1.06	38.94±1.00	38.83±0.88
	Harmonious marital status (%)		100	100	100	100
	Toxic exposure history (%)		0	0	0	0
	Radioactive material exposure history (%)		0	0	0	0
	Gravida (times)	*	1.65±0.79	1.97±1.01	2.86±1.48	4.02±1.66
	Pregnancy (times)	*	1.35±0.61	1.48±0.51	2.06±0.71	2.02±0.56
	History of abortion (times)	*	0.29±0.47	0.48±0.71	0.83±1.03	2.02±1.52
	History of preterm birth (times)		0	0	0.03±0.17	0
	Family history of type 2 diabetes mellitus (%)		0	0	0	2.08
	History of other diseases (%)		0	0	0	0
Physical Examination Information	Body mass index (kg/m2)	*	26.98±4.13	25.53±2.96	27.11±3.08	27.71±3.38
	Blood type O (%)		17.65	40.63	36.11	33.33
	Body temperature (℃)		36.86±0.23	36.81±0.24	36.73±0.52	36.80±0.21
	Pulse (times/min)		90.24±12.53	86.85±9.55	88.94±7.53	86.33±9.29
	Fetal heart rate (times/min)		140.94±7.21	141.48±3.44	142.83±3.47	143.10±3.57
	Systolic blood pressure (mmHg)		114.94±9.32	113.91±8.17	111.72±11.67	113.25±10.80
	Diastolic blood pressure (mmHg)		73.94±7.91	73.55±6.10	75.94±7.26	74.73±8.01
	Fundus high (cm)		35.59±2.12	34.12±2.65	34.78±2.00	34.73±2.17
	Interspinous diameter of anterior superior iliac (cm)		23.59±0.51	23.91±0.63	23.56±0.56	23.83±0.75
	Diameter between iliac crest (cm)		26±0	25.88±1.98	26±0.24	26.17±0.75
	The sacral disgrace outside diameter (cm)		18.88±0.49	19±0	18.94±0.23	18.93±0.29
	Intertubercular diameter of ischium (cm)		9.03±0.12	9±0	8.96±0.18	8.98±0.21
	Abdomen circumference (cm)		98.47±7.37	98.15±6.67	100.26±6.49	100.63±6.09
Medications	Probiotic supplement (%)		0	0	0	0
	Pharmacohistory (%)		0	0	0	0
	History of drug allergy (%)		0	0	2.777777778	0
Dietary habits	Food allergies (%)		0	0	0	0
	Smoking (%)		0	0	0	0
	Drinking alcohol (%)		0	0	0	0
Newborn Physical Examination	Previous female fetuses (%)		40	62.5	58.62	57.14
	Previous fetus weight (g)		3115.9±579.41	3173.44±555.69	3318.45±421.49	3319.74±452.25
	Female fetuses (%)		41.18	36.36	47.22	43.75
	Fetal body length (cm)	*	50.24±0.83	49.91±0.58	49.96±0.64	50.31±0.75
	Fetus weight (g)		3527.06±451.67	3361.67±451.96	3376.53±412.03	3526.98±480.35
	Apgar Score		9.94±0.24	9.82±0.64	9.97±0.17	9.96±0.20

Supplemental Table S1. **General clinical information of participants.** * denotes FDR < 0.05.

Category	Variables	20-25 years (N=17)	25-30 years (N=33)	30-35 years (N=36)	⩾35 years (N=48)
Thyroid related indicators	All T4 (ng/ml)	81.51±21.42	92.63±17.17	87.72±13.20	87.09±17.98
	Free T3 (pmol/L)	4.64±0.69	4.67±0.58	4.42±0.58	4.36±0.67
	Free T4 (pmol/L)	10.6±2.09	11.43±3.52	9.97±2.70	10.42±2.83
	Thyroid stimulating hormone (mIU/L)	2.82±2.05	2.83±1.38	2.06±0.99	2.21±1.22
	Thyroid peroxidase antibody (IU/ml)	5.84±5.19	14.04±14.49	16.50±26.99	11.41±14.91
Coagulation related indicators	Prothrombin time (s)	11.12±0.39	11.07±0.44	11.21±0.51	11.16±0.59
	International normalized ratio	0.94±0.03	0.94±0.04	0.95±0.04	0.95±0.05
	Activated partial thromboplastin time (s)	27.26±1.71	27.94±1.64	27.61±1.94	27.78±2.11
	Thrombin time (s)	14.06±1.01	13.55±1.07	13.40±0.88	13.50±0.81
	Fibrinogen (g/L)	4.21±0.58	4.35±0.52	4.33±0.53	4.33±0.44
	D-dimer (mg/L)	1.99±1.00	2.29±1.42	1.84±1.32	1.96±1.21
	Degradation products of fibrin (mg/L)	5.32±2.35	5.44±3.03	4.40±2.92	4.85±2.80
Urine routines	Clear transparency (%)	92.31	90	96.67	92.86
	Protein negative (%)	100	73.33	76.67	69.05
	Glucose negative (%)	92.31	83.33	96.67	95.24
	Ketone negative (%)	100	86.67	93.33	78.57
	Bilirubin negative (%)	100	100	100	100
	Urobilinogen negative (%)	100	96.67	96.67	95.24
	Occult blood negative (%)	92.31	83.33	90	78.57
	Leucocyte negative (%)	69.23	50	46.67	59.52
	Nitrite (%)	100	100	100	97.62
	Vitamin C (%)	53.85	80	73.33	73.81
	Sperm (%)	100	100	100	100
	RBCI (%)	100	100	100	100
	Tube (%)	100	93.33	96.67	97.62
	The pathological type tube (%)	100	100	96.67	97.62
	Density	1.02±0.01	1.02±0.01	1.02±0.01	1.02±0.01
	Pondus hydrogenii value	6.58±0.53	6.6±0.56	6.56±0.74	6.39±0.51
	Red blood cell (%)	7.77±13.97	137.83±729.66	5.33±4.87	11.43±35.14
	White blood cell count (%)	36.62±104.61	33.83±99.11	43.33±122.61	17.07±32.15
	Epithelial cell count (%)	21.85±19.65	43.5±65.15	52.83±49.60	37±61.84
	Small round epithelial cell (%)	0.77±1.01	0.77±1.19	1.03±1.19	1.07±1.69
	Viscose silk (%)	23.85±34.92	29.8±39.83	38.97±60.81	51.14±81.84
	Fungus (%)	0±0	0±0	0.2±1.10	0.05±0.31
	Crystal (%)	18.69±28.67	6±17.99	26.13±64.00	10.21±34.06

Supplemental Table S2. Thyroid related indicators, and coagulation-related indicators and urine routine indicators information of participants in different age groups.

ID	Genus	R	NUll_*P* value
Absolute value of monocytes	*Corynebacterium*	-0.258	0.0106
	Facklamia	0.189	0.0354
	Peptococcus	-0.1999	0.0382
	Bulleidia	-0.1918	0.0449
Absolute neutrophil count	*Corynebacterium*	-0.2347	0.0183
	Facklamia	0.2045	0.0257
	Enterococcus	-0.2165	0.0272
	Methylotenera	-0.1961	0.0413
	Dorea	0.1763	0.0456
	Candidatus Koribacter	-0.1864	0.0498
White blood cell count	*Corynebacterium*	-0.233	0.019
	Facklamia	0.2069	0.0244
	Enterococcus	-0.2167	0.027
	Dorea	0.177	0.045
	Candidatus Koribacter	-0.1905	0.046
Platelet distribution width	*Butyricimonas*	0.2531	0.0083
	Rothia	0.2217	0.0175
	Mogibacterium	0.2066	0.0245
	Candidatus Arthromitus	-0.2161	0.0274
	Alloiococcus	0.201	0.0277
	Adlercreutzia	0.2	0.0283
	Oscillospira	0.1916	0.0336
	Campylobacter	0.1849	0.0385
	Phascolarctobacterium	0.184	0.0392
	Odoribacter	0.182	0.0408
	Vibrio	-0.1936	0.0434
	Lachnospira	0.1756	0.0462
	Flavobacterium	-0.189	0.0474
	Ruminococcus	0.1718	0.0497
Age	*Slackia*	0.2784	0.0043
	Haemophilus	0.2672	0.0058
	Neisseria	0.2438	0.0104
	Bulleidia	0.234	0.0132
	Anaerostipes	0.2036	0.0261
	Streptomyces	0.2031	0.0264
	Vibrio	0.2025	0.0268
	Parabacteroides	0.1764	0.0454
	Acinetobacter	0.1764	0.0455
	Candidatus Arthromitus	0.1763	0.0456
	Methylotenera	0.1724	0.0491
Body mass index	*Staphylococcus*	-0.2642	0.0091
	Sphingobium	0.2087	0.0234
	Faecalibacterium	0.189	0.0354
	Veillonella	-0.2008	0.0376
	Propionibacterium	-0.1906	0.0459
	Alloiococcus	0.1743	0.0473
Number of gravida	*Parvimonas*	-0.2074	0.0329
	cc_115	-0.1947	0.0424
	Pseudonocardia	0.1785	0.0437
	Granulicatella	0.1729	0.0487
History of abortion	*Pseudonocardia*	0.2046	0.0256
	Granulicatella	0.2024	0.0268
	Mogibacterium	-0.1897	0.0467
Number of pregnancy	*cc_115*	-0.2252	0.0225
	Acinetobacter	0.2101	0.0227
	Parvimonas	-0.211	0.0305
	Escherichia	-0.2109	0.0306

Supplemental Table S3. The associations for each pair of clinical indicator and gut microbe in mothers.

A

Group (years)	Mother	Offspring	Paired
20-25	10	17	10
25-30	33	34	33
30-35	36	35	34
≥35	40	47	39

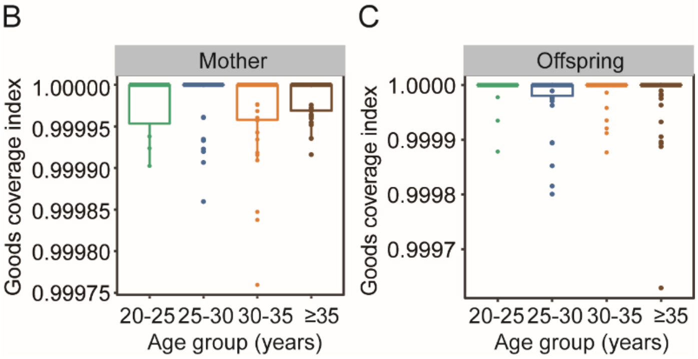

Supplemental Figure S1. Number of samples and data size of 16S rRNA gene sequencing in this study. A. Number of feces and meconium samples collected in this study. Among the samples, paired samples refer to the feces of the parturient and the meconium of the newborns delivered by the parturient. B. Goods coverage index of maternal gut microbiota in this study. C. Goods coverage index of newborns meconium in this study.

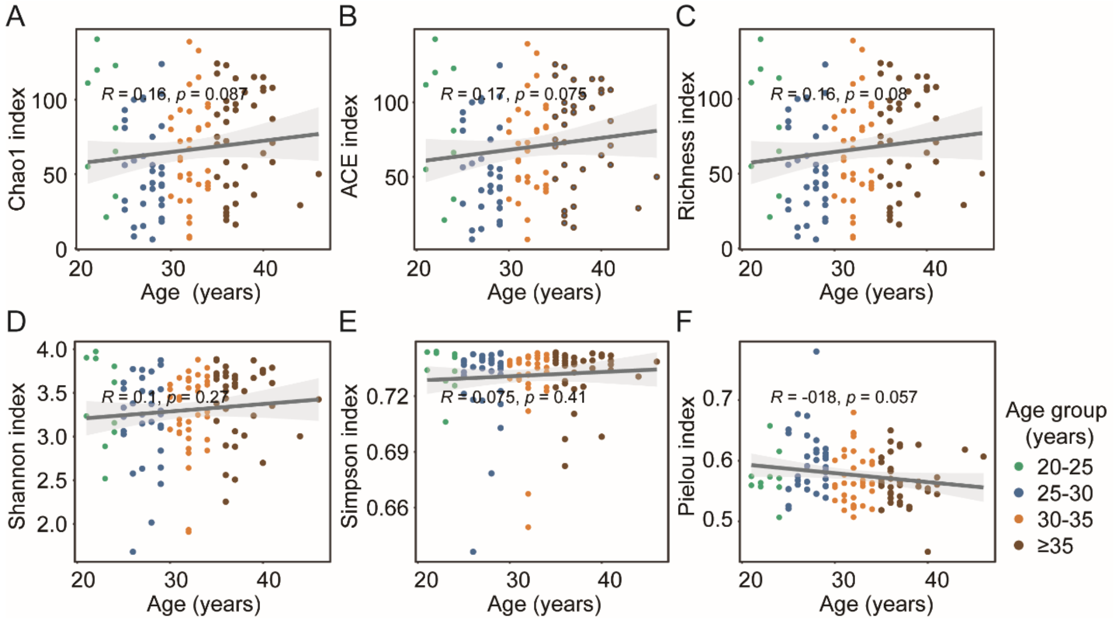

Supplemental Figure S2. **The correlation between age and alpha diversity in maternal gut microbiota.** A. Correlation of Chao1 index with age. B. Correlation of ACE index with age. C. Correlation of richness index with age. D. Correlation of Shannon index with age. E. Correlation of Simpson index with age. F. Correlation of Pielou index with age. Correlation was calculated by Spearman method.

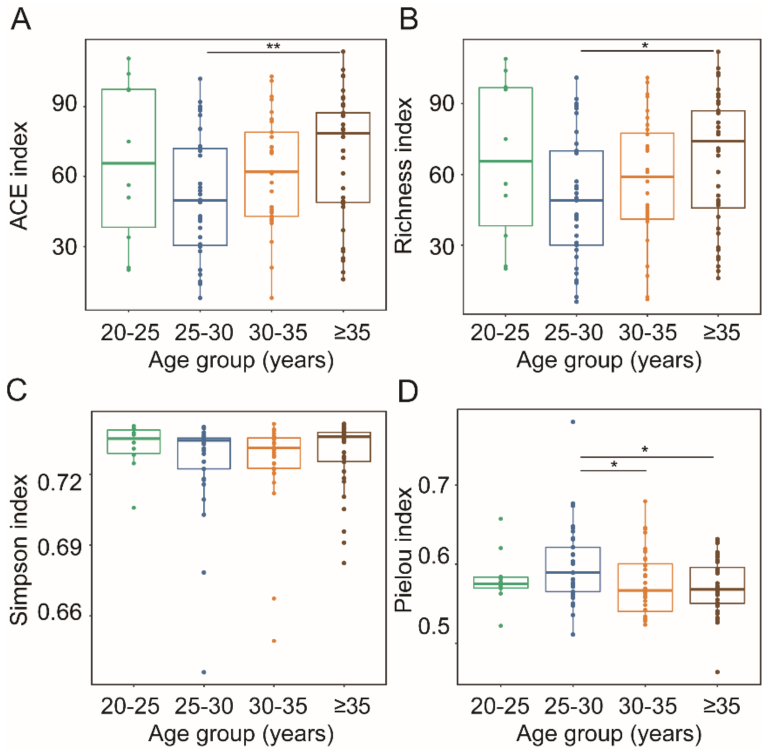

Supplemental Figure S3. **Comparison of α-diversity among different age groups in maternal gut microbiota.** A. Correlation of ACE index with age. B. Correlation of richness index with age. C. Correlation of Simpson index with age. D. Correlation of Pielou index with age. * denotes $P < 0.05$; ** denotes $P < 0.01$.

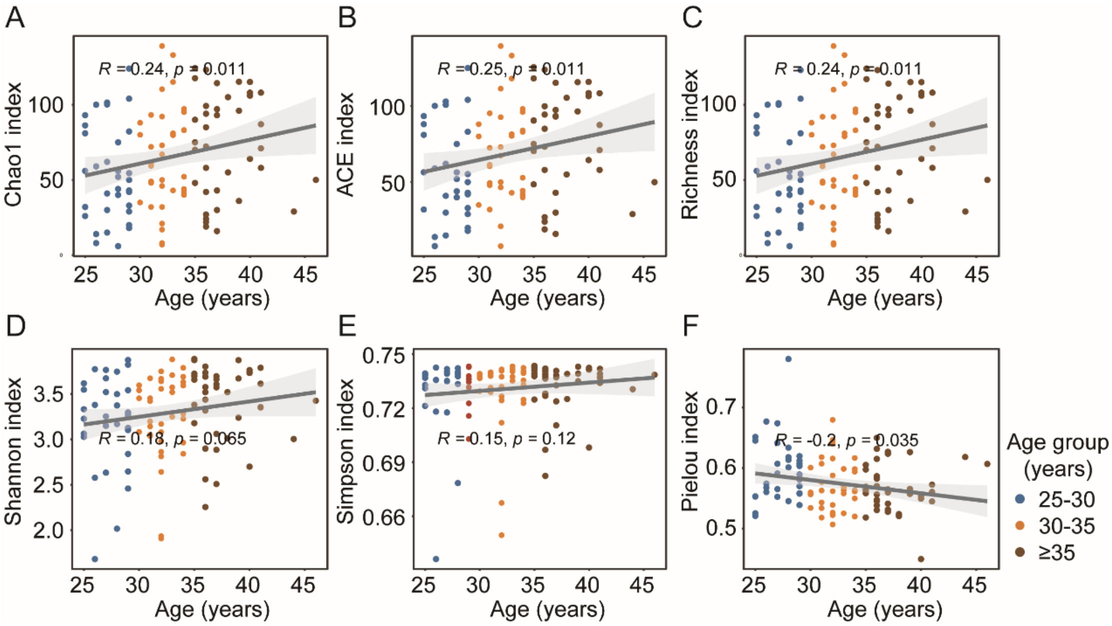

Supplemental Figure S4. **The correlation between age and alpha diversity in maternal microbiota.** A. Correlation of Chao1 index with age. B. Correlation of ACE index with age. C. Correlation of richness index with age. D. Correlation of Shannon index with age. E. Correlation of Simpson index with age. F. Correlation of Pielou index with age. Correlation was calculated by Spearman method.

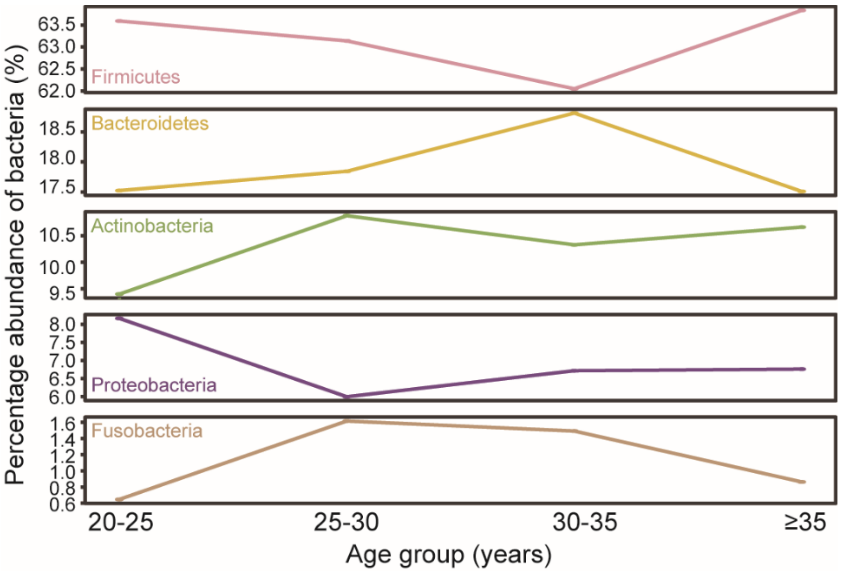

Supplemental Figure S5. The top 5 most abundant phyla in maternal microbiota of different age groups.

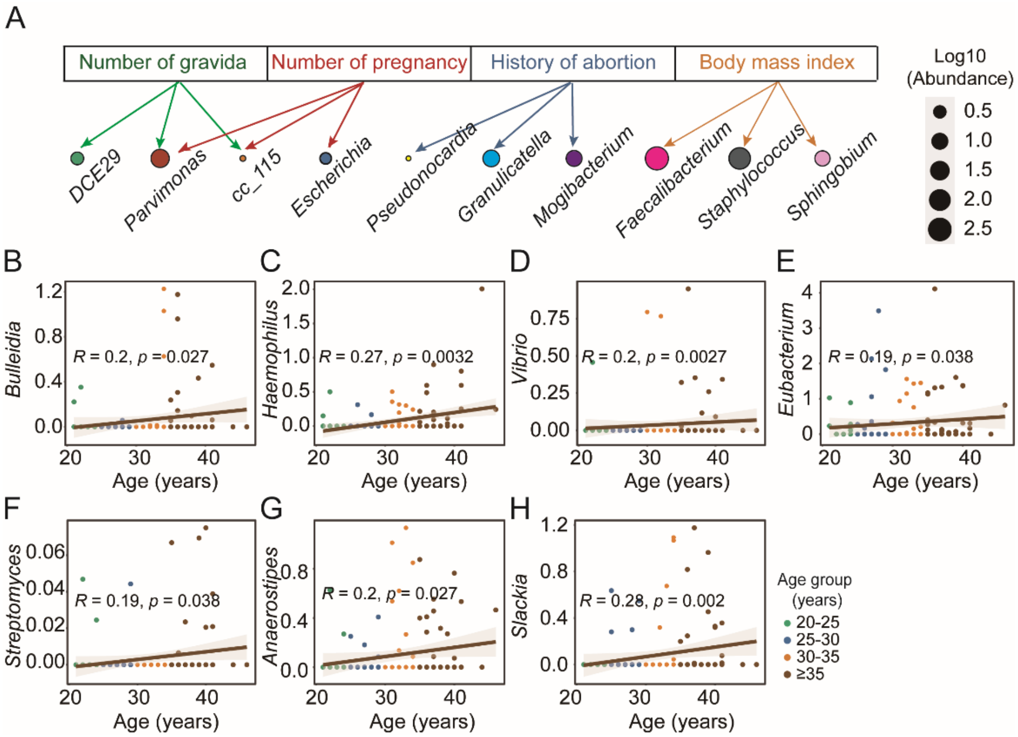

Supplemental Figure S6. The correlation between general clinical data and bacteria at the genus level in maternal gut microbiota. A. Bacteria that are significantly associated with general clinical data with significant differences in age groups, the circle size represents the log10 (average abundance) of the bacteria. B. Correlation of *Bulleidia* with age. C. Correlation of *Haemophilus* with age. D. Correlation of *Vibrio* with age. E. Correlation of *Eubacterium* with age. F. Correlation of *Streptomyces* with age. G. Correlation of *Anaerostipes* with age. H. Correlation of *Slackia* with age. The correlation was calculated by Spearman method.

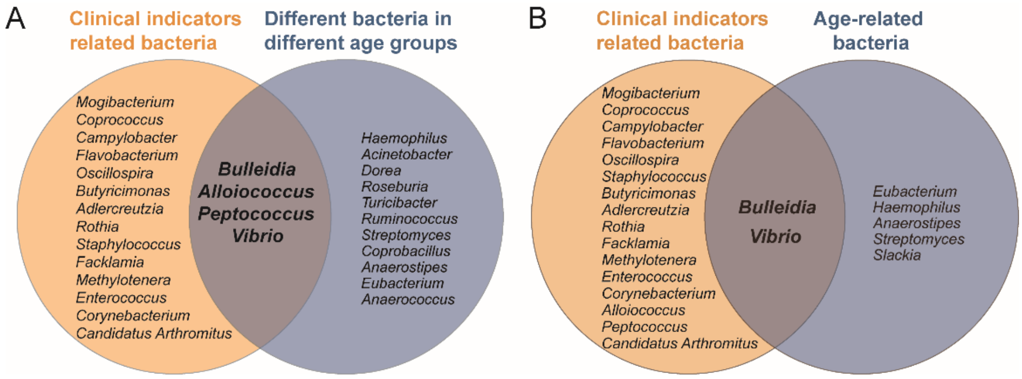

Supplemental Figure S7. All bacteria that have significant different with both clinical indicators and age in the mother. A. All bacteria that have significant correlation with clinical indicators and have significant different in different age groups. B. All bacteria that have significant correlation with clinical indicators and age.

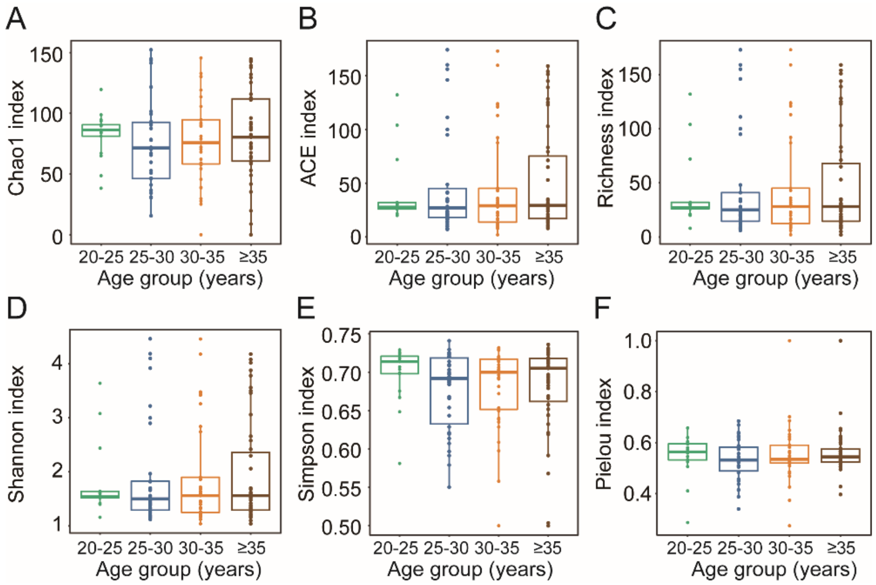

Supplemental Figure S8. Comparison of α diversity among different age groups in neonatal meconium. A. Comparison of Chao1 index in different age groups. B. Comparison of ACE index in different age groups. C. Comparison of richness index in different age groups. D. Comparison of Shannon index in different age groups. E. Comparison of Simpson index in different age groups. F. Comparison of Pielou index in different age groups.

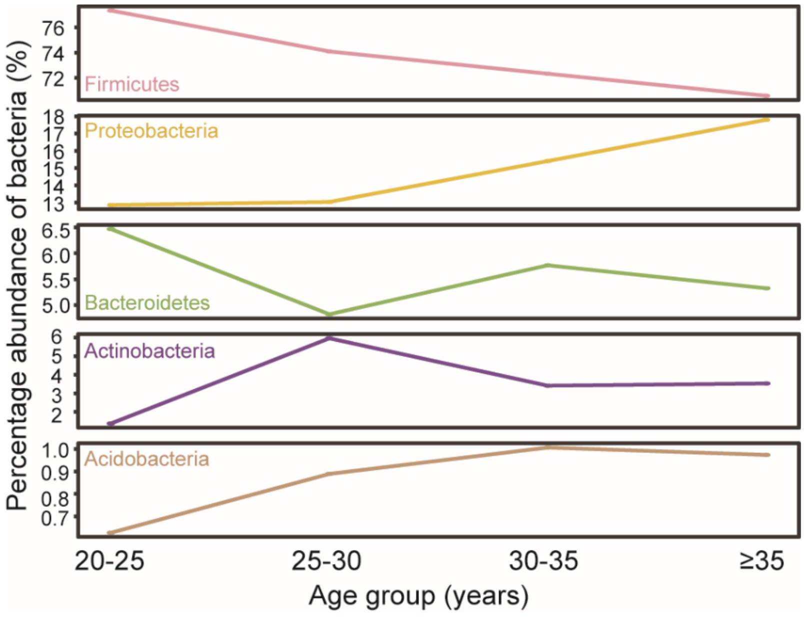

Supplemental Figure S9. The top 5 most abundant bacterial phyla in newborn microbiota in different age groups.

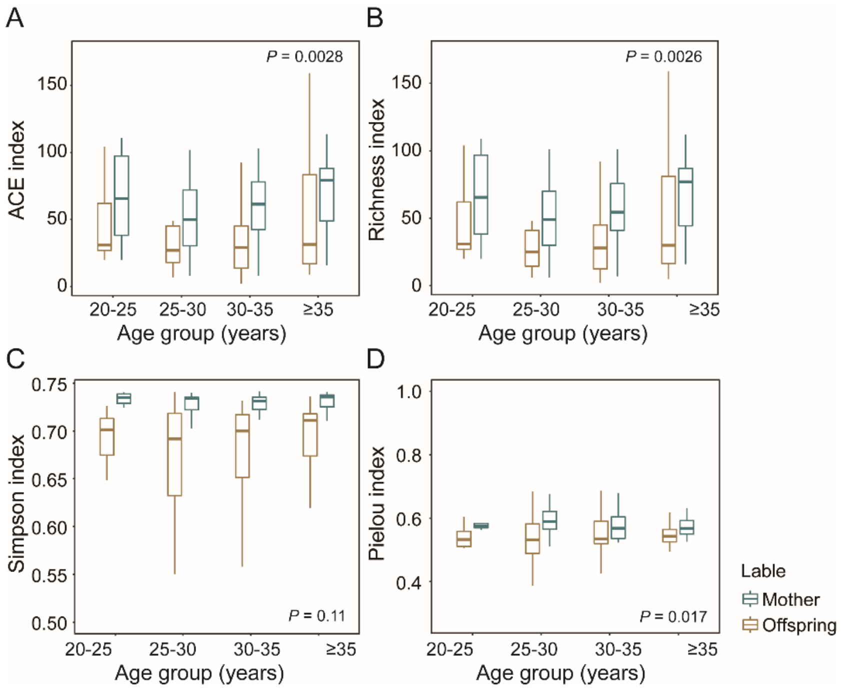

Supplemental Figure S10. Comparison of α-diversity of bacterial communities among different age groups in paired maternal feces and neonatal meconium samples. A. Comparison of ACE index of bacterial communities among different age groups in paired maternal feces and neonatal meconium samples. B. Comparison of Richness index of bacterial communities among different age groups in paired maternal feces and neonatal meconium samples. C. Comparison of Simpson index of bacterial communities among different age groups in paired maternal feces and neonatal meconium samples. D. Comparison of Pielou index of bacterial communities among different age groups in paired maternal feces and neonatal meconium samples.

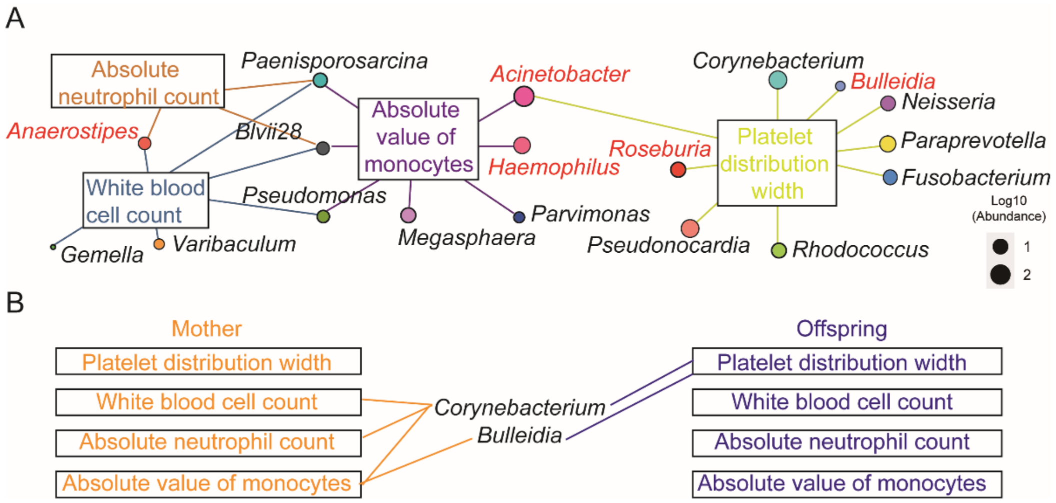

Supplemental Figure S11. The clinical physiological and biochemical indices related to intestinal microbes of newborns. A. All bacteria that have significant correlation with clinical test indicators, total protein, creatinine, platelet distribution width, white blood cell count, absolute neutrophil count, absolute value of monocytes indicators that are significantly related to age group. Bacteria marked in red text represents bacteria with significant differences in maternal microbiota among different age groups. B. Identical bacteria that have significant correlation with clinical physiological and biochemical indicators in both mother and newborn.
